# Response Prediction of Chemoradiotherapy for Rectal Cancer Using Rapid Semi-Automated Flow Cytometry

**DOI:** 10.3390/cancers18010001

**Published:** 2025-12-19

**Authors:** Hiroyuki Amagai, Koichi Hayano, Takahiro Shioyama, Akane Suzuki, Gaku Ohira, Tetsuro Maruyama, Toru Tochigi, Koichiro Okada, Takahiro Arasawa, Nobufumi Sekino, Ryoya Mizumachi, Soichiro Hirasawa, Masaya Uesato, Michihiro Maruyama, Yoshihiro Kurata, Atsushi Hirata, Hisahiro Matsubara

**Affiliations:** 1 Department of Frontier Surgery, Graduate School of Medicine, Chiba University, Chiba City 260-8670, Japan; k-hayano@chiba-u.jp (K.H.); ohira@faculty.chiba-u.jp (G.O.); t.maruyama@chiba-u.jp (T.M.); tochigi@chiba-u.jp (T.T.); kookada@chiba-u.jp (K.O.); t-arasawa@hotmail.co.jp (T.A.); n.sekino@chiba-u.jp (N.S.); firebolt227@yahoo.co.jp (R.M.); shirasawa@chiba-cc.jp (S.H.); uesato@faculty.chiba-u.jp (M.U.); michi-maruyama@chiba-u.jp (M.M.); kuratayoshihiro@yahoo.co.jp (Y.K.); a.hirata@chiba-u.jp (A.H.); matsuhm@faculty.chiba-u.jp (H.M.); 2 Nihon Kohden Corporation, Tokyo 161-8560, Japan; takahiro_shioyama@mb1.nkc.co.jp (T.S.); akane_suzuki@mb1.nkc.co.jp (A.S.)

**Keywords:** rectal cancer, flow cytometry, chemoradiation

## Abstract

(1) Background: The effectiveness of chemoradiotherapy (CRT) for rectal cancer varies greatly among patients, and no reliable biomarker for predicting treatment response currently exists. We investigated whether cell cycle analysis using rapid semi-automated flow cytometry could predict the effectiveness of CRT. (2) Methods: This prospective study included 32 patients with rectal cancer undergoing CRT. The cell cycle of biopsy specimens from rectal cancer patients before CRT was semi-automatically and rapidly analyzed using Celltac PEAK as flow cytometer. The proportion of cell cycle phases is compared with treatment response after CRT. (3) Results: Patients with a tumor reduction rate ≥ 30% on CT showed a significantly higher proportion of cells in the Over G2/M phase compared with those with <30% reduction (median 2.45% vs. 0.95%, *p* = 0.022). Similarly, tumors demonstrating disappearance of FDG uptake after CRT had a significantly higher proportion of cells in the Over G2/M phase compared with those with persistent FDG uptake (median 4.05% vs. 1.24%, *p* = 0.024). (4) Conclusions: A high proportion of cells in the Over G2/M phase could be a potential biomarker for predicting tumor shrinkage in CRT for rectal cancer.

## Simple Summary

There is no reliable biomarker for predicting the effectiveness of CRT in rectal cancer. The aim of our study is to assess the potential value of cell cycle analysis by rapid semi-automated flow cytometry to predict the effectiveness of CRT. Thirty-two patients with rectal cancer who underwent CRT were enrolled in this study. The cell cycles of biopsy specimens from rectal cancer before CRT are analyzed using Celltac PEAK as flow cytometer. The group with a reduction rate of 30% or more in CT has a significantly higher proportion of cells in the Over G2/M phase. The group with PET accumulation disappearance on 18F-FDG PET/CT has significantly higher proportion of Over G2/M as well. A high proportion of cells in the Over G2/M phase could be a potential biomarker for tumor shrinkage effects in CRT for rectal cancer.

## 1. Introduction

Preoperative chemoradiotherapy (CRT) followed by surgery for rectal cancer is becoming increasingly common in Japan [1], and in the 2024 edition of the guidelines, preoperative chemoradiotherapy is weakly recommended for rectal cancer with a high risk of local recurrence [2]. The local control rate of CRT for rectal cancer is high [3,4,5], and it was reported that CRT enabled conversion surgery or organ preservation in initially unresectable advanced rectal cancer [6]. In some hospitals, total neoadjuvant therapy, which combines chemotherapy with chemoradiotherapy, is being initiated [7,8]. On the other hand, there are cases where CRT is ineffective, remote metastasis occurs, or adverse events arise. If we could easily predict the effectiveness of CRT before treatment, it would enable tailored medical care for each individual case, providing the optimal treatment. Therefore, the development of biomarkers for predicting the effectiveness of CRT is really needed.

On the other hand, it was reported that the state of the cell cycle was related to radiation sensitivity [9]. The cell cycle is analyzed by flow cytometry, but the procedure is cumbersome, and variability among examiners is a concern [10]. The Celltac PEAK is characterized by its ability to analyze the cell cycle semi-automatically. If the cell cycle could be conveniently measured using Celltac PEAK with specimens from endoscopic biopsies, it might be possible to utilize it for predicting the effectiveness of CRT.

Therefore, the objective of this study is to investigate whether the cell cycle of pretherapeutic endoscopic biopsy specimens analyzed by the Celltac PEAK can predict the treatment response to CRT for rectal cancer.

## 2. Materials and Methods

### 2.1. Study Population

This prospective study included patients with pathologically proven locally advanced rectal cancers who were treated with neoadjuvant CRT at our hospital between 2017 and 2022. Because this study focused on the local treatment response to CRT, we included patients who did not undergo surgery after completion of CRT for any reason. The indication of CRT at our institution was defined as follows: rectal cancers with the tumor edge located below the peritoneal reflection and infiltrating beyond the muscularis propria (cT3-T4) or those with lymph node metastasis. Patients with distant metastases were excluded from this study. This study was approved by the Clinical Research Ethics Committees of Chiba University and was registered in the University Hospital Medical Information Network (Umin) Clinical Trials Registry as UMIN 000031130 (http://www.umin.ac.jp/ctr/index.htm, accessed on 10 December 2025). All procedures performed in the study were in accordance with the 1964 Helsinki declaration and its later amendments.

### 2.2. Flow Cytometry

**Figure 1:**
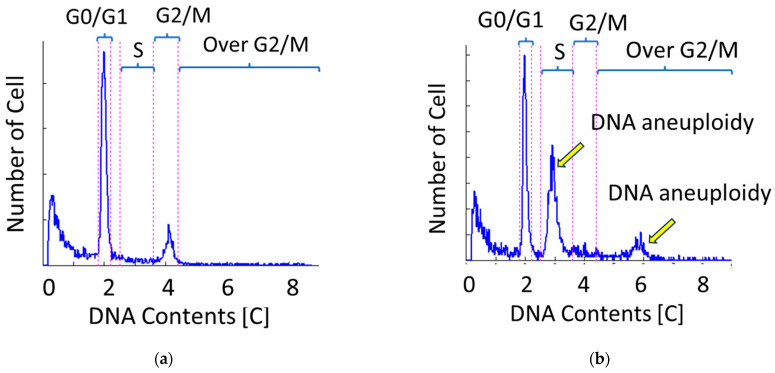
DNA histogram. (**a**) A case without aneuploidy. (**b**) A case with aneuploidy. Aneuploid is defined as a cell population clearly separated from G0/G1 or G2/M phase.

Before CRT, a 2 mm-sized piece of tissue was taken from the tumor center by lower gastrointestinal endoscopy, and the nuclear content of cells was semi-automatically and rapidly measured using the Celltac PEAK (FCM-2200; Nihon Kohden Corporation, Tokyo, Japan). The methodology for flow cytometry using Celltac PEAK is consistent with the previous report [11]. Briefly, specimens were placed in a microtube and immersed in a staining reagent kit (DNA PEAK, FC-220V; Nihon Kohden Corporation, Tokyo, Japan) that included ribonuclease A, TritonX-100, and propidium iodide. The specimen was then disrupted by repetitive pipetting for 200 s with an automatic cell isolation system for flow cytometry consisting of a cell isolation unit and a staining reagent kit prototype device (Nihon Kohden Corporation, Tokyo, Japan). Cell cycle analysis was performed based on nuclear content, and the proportion of cells in each cell cycle phase (G0/G1 phase, S phase, G2/M phase, and Over G2/M phase) is measured. DNA index is defined as the ratio of test (tumor) sample/standard DNA contents. According to the previously reported definition [12], the Malignancy Index (MI) was defined as the ratio of the number of cells with greater-than-normal DNA content to the total number of cells (N); that is, MI = (S + G2/M + Over G2/M)/N. Ploidy analysis with flow cytometry can reveal the DNA heterogeneity of cells. DNA aneuploidy was seen on the histogram as a different peak from the diploidy peak (Figure 1).

### 2.3. Response Evaluation

Prior to CRT, imaging evaluations including CT, 18F-FDG PET/CT, and lower gastrointestinal endoscopy were conducted. The assessment of treatment efficacy after CRT was performed 2–3 weeks after the completion of radiation therapy using CT, 18F-FDG PET/CT, and lower gastrointestinal endoscopy. Three patients who underwent consolidation chemotherapy were evaluated for their treatment response of CRT before initiating consolidation chemotherapy. CT scans were conducted axially, measuring the maximum thickness of the tumor’s most prominent rim. The measurement was taken from one side of the rim. After CRT, the thickness was assessed at the same location to ensure consistency. In CT, the group with tumor shrinkage rate of 30% or more before and after CRT was classified as “CT responder”, while the group with less than 30% shrinkage was classified as “CT non-responder”. In 18F-FDG PET/CT, the group in which the maximum standardized uptake value (SUV max) decreased to less than 5.0 after CRT was classified as “Disappearance of PET accumulation,” while the group with SUV max equal to or above 5.0 was classified as “Persistence of PET accumulation.” The reason for assessing efficacy based on the disappearance of PET accumulation after CRT was that 18F-FDG PET/CT was not performed before CRT in five cases. The cutoff value for PET uptake disappearance was set at an SUVmax of 5.0, based on the previous study [13].

After completion of CRT, radical surgery was performed 6–8 weeks later, and the pathological therapeutic effect of CRT was evaluated with the use of surgical specimens. The histological assessment of the therapeutic effect of CRT on the primary tumor was conducted based on the following criteria: Grade 3, indicating the absence of viable carcinoma cells in the main tumor (pCR); Grade 2, where less than 1/3 of the residual cancer cells were viable; Grade 1, where more than 1/3 of cancer cells were observed; and Grade 0, indicating no effect was found. Grade 2 and 3 groups were categorized as histological responder, while Grade 0 and 1 groups were categorized as histological non-responder.

### 2.4. Statistical Analysis

All statistical analyses were performed using JMP Pro software, version 14.2.0 (SAS Institute Inc., Cary, NC, USA). Continuous variables were summarized as medians with interquartile ranges (IQRs), and categorical variables as absolute frequencies and percentages. Focusing on the cell cycle, patients were stratified into high and low Over G2/M phase groups, and baseline demographics were characterized accordingly. Group comparisons were conducted using the Wilcoxon rank-sum test for cell cycle phase distributions and Fisher’s exact test for aneuploidy. Optimal cut-off values for continuous predictors were determined by receiver operating characteristic (ROC) curve analysis, with predictive accuracy assessed by the area under the ROC curve (AUC). Recurrence-free survival (RFS) was estimated using the Kaplan–Meier method, and differences between groups were evaluated with the log-rank test. Cox proportional hazards regression models were applied to derive hazard ratios (HRs) and 95% confidence intervals for RFS in relation to clinicopathological variables; however, only univariable analyses were performed, and multivariable modeling was not conducted. A two-sided *p* value < 0.05 was considered statistically significant.

## 3. Results

### 3.1. Patient Characteristics

Forty-two rectal cancer patients underwent biopsy prior to initiation of treatment in order to obtain specimens for flow cytometry at Chiba University Hospital between April 2017 and June 2022. However, 10 cases did not undergo CRT because they were treated with surgery alone or chemotherapy; a total of 32 cases who underwent neoadjuvant CRT (nCRT) were eligible for this study.

Patients’ characteristics are presented in Table 1. For the entire cohort, the mean age was 62.9 years (SD = 11.0) and 23/32 were male (71.9%). All patients enrolled in this study were of Asian ethnicity. Location of the tumor was Rectosigmoid: Upper rectum: Lower rectum = 1:8:23. Median size of maximum tumor diameter was 4.6 cm (IQR, 3.9–5.7). CRT was completed in all 32 cases. All patients underwent CT for CRT efficacy assessment. In total, 30 patients underwent 18F-FDG PET/CT, and 2 patients did not due to unknown reasons. For 30 patients, surgery after CRT was performed, and for 2 patients, surgery was canceled because of the emergence of new unresectable metastasis after CRT. R0 surgery was achieved in all patients who underwent surgery. RECIST (v1.1 criteria) was CR:PR:SD:PD = 2:24:5:1. The median postoperative follow-up period was 40.2 (IQR, 16.1–59.7) months, with eight deaths during that period (five deaths from cancer and the remaining three deaths from complications such as pneumonia, lymphoma, or glioblastoma). Cancer recurrence was observed in 11 cases (5 cases had lung metastasis, 2 had lymph node metastasis, 2 had liver metastasis, 1 had bone metastasis, and 1 had local recurrence). Two cases in which remote metastasis occurred after CRT and surgery was canceled were considered with the recurrence day designated as day 0.

### 3.2. Clinical Response

**Figure 2:**
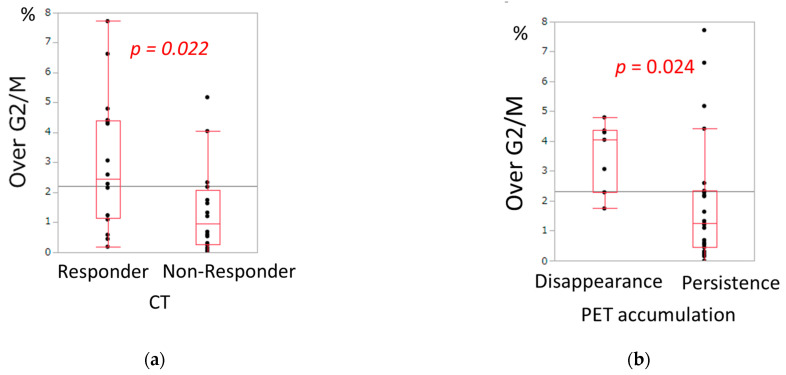
(**a**) Relationship between tumor shrinkage in CT and the proportion of Over G2/M phase. (**b**) Relationship between PET accumulation and the proportion of Over G2/M phase.

Cell cycle, DNA Index, Malignancy Index, and aneuploidy were compared with the clinical response. The proportion of Over G2/M was significantly higher in CT responders than in CT non-responders (*p* = 0.022, Table 2, Figure 2a). The proportion of Over G2/M was also significantly higher in disappearance of PET accumulation than in persistence of PET accumulation (*p* = 0.024, Table 3, Figure 2b). Although the difference was not statistically significant, the group with disappearance of PET uptake tended to have a higher Malignancy Index compared to the group with persistent uptake (*p* = 0.101, Table 3). Regarding cell cycle, DNA index, Malignancy Index, or aneuploidy, there were no significant differences between histological responders and histological non-responders (Table 4).

### 3.3. Recurrence-Free Survival

**Figure 3:**
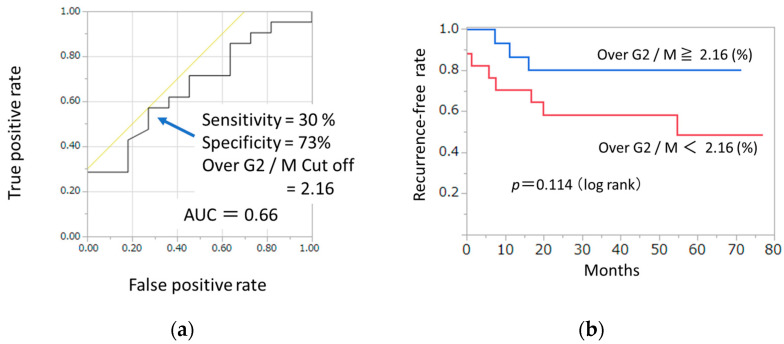
(**a**) Receiver operating characteristic (ROC) curve for the relationship between 3-year recurrence and the proportion of Over G2/M phase. The area under the curve (AUC) is 0.66. The sensitivity was 30% and specificity was 73% at a cutoff value of 2.16. (**b**) Relationship of proportion of Over G2/M and recurrence free survival. The Kaplan–Meier curve demonstrated that the group of Over G2/M ≥ 2.16% tended to have better relapse-free survival, but this tendency was not statistically significant (*p* = 0.114).

To assess the discriminatory power of Over G2/M for distinguishing 3-year recurrence from 3-year non-recurrence revealed an AUC of 0.66. Based on the Youden index, the optimal cut-off value of the Over G2/M ratio was 2.16, yielding a sensitivity of 0.30 and a specificity of 0.73. (Figure 3a). In Kaplan–Meier analysis, patients with tumors having the cells of Over G2/M ≥ 2.16% tended to show a better recurrence-free survival (RFS) compared to those having the cells of Over G2/M < 2.16%, but this tendency was not significant, (*p* = 0.114, 3-year RFS; 80% vs. 58%, Figure 3b). Since two case in the Over G2/M < 2.16% group was found to have recurrence before the completion of CRT, the starting point for this group was set at 0.88. Although Kaplan–Meier analysis did not demonstrate a statistically significant difference, patients with higher Over G2/M values tended to show better recurrence-free survival. To further clarify the prognostic impact of Over G2/M and other clinicopathologic variables, Cox proportional hazards regression analysis was performed, and the hazard ratios for recurrence-free survival are summarized in Table 5. Positive lymph node metastasis was significantly associated with poorer recurrence-free survival, with a hazard ratio of 5.23. In contrast, the group with Over G2/M ≥ 2.16% demonstrated a hazard ratio of 0.76, and although the difference did not reach statistical significance (*p* = 0.11), a trend toward improved outcomes was observed. Following the analysis of hazard ratios for recurrence-free survival according to clinicopathologic variables, we next compared patient demographics between the Over G2/M ≥ 2.16% group and the Over G2/M < 2.16% group to further characterize the clinical background associated with this cutoff (Table 1). In the comparison of patient demographics, the Over G2/M ≥ 2.16% group demonstrated significantly lower levels of CA19-9. In contrast, this group exhibited a significantly higher Malignancy Index and a greater frequency of aneuploidy compared with the G2/M < 2.16% group. Given the relatively small sample size, the reliability of the survival analysis is limited. Nevertheless, Kaplan–Meier and Cox proportional hazards regression analyses were performed to explore the potential prognostic impact of Over G2/M.

## 4. Discussion

CRT for advanced rectal cancer is rapidly gaining popularity with the aim of improving local control rates. Complete remission is achieved in approximately 10–60% of cases [14], and in some institutions, a “watch and wait” strategy is also adopted for patients achieving a clinical complete response [15]. However, ineffective CRT may lead to loss of curative treatment opportunities and an increased risk of distant metastasis. In addition, there is also the issue of adverse events associated with CRT [16,17]. Thus, reliable biomarkers to predict CRT efficacy are urgently needed.

Although several attempts have been made to predict tumor response to CRT using a variety of clinical and pathological features [18], no validated prediction model has yet been established for clinical practice [19]. On the other hand, since the cell cycle is closely related to radiosensitivity and chemosensitivity [9,20], rapid and simple measurement of cell cycle distribution in pretreatment biopsy specimens may provide a novel and practical predictive tool for CRT in rectal cancer. Although studies of cell cycle profiles and aneuploidy using flow cytometry date back to the 1990s [21], clinical application has been limited due to technical variability and time-consuming procedures. To overcome these limitations, Mimura et al. developed a novel cell preparation system to facilitate highly accurate and reproducible measurements, with the aim of promoting the clinical application of aneuploidy analysis [11,22]. Their research led to the development of Celltac PEAK, an automated flow cytometer. Celltac PEAK automatically stains the nuclei of isolated cells from tissues and measures them using flow cytometry. It takes only 10 min and it makes it possible to analyze cell cycle and aneuploid [11]. Previous reports suggest that Celltac PEAK can aid in the diagnosis of gastrointestinal stromal tumors [23] and may predict both malignancy grade and radiosensitivity in glioblastoma [12].

In the present study, the group with Over G2/M ≥ 2.16% was significantly associated with a higher frequency of aneuploidy (Table 1). Many previous studies on the relationship between the cell cycle and radiosensitivity have focused on aneuploidy [21]. High radiosensitivity of aneuploid tumors has been described in various malignancies, including rectal cancer [24], bladder cancer [25,26], oral cavity squamous cell carcinoma [27], cervical cancer [28], and esophageal cancer [29]. In contrast, our study found no significant association between aneuploidy and either CT tumor shrinkage or 18F-FDG PET/CT response. Instead, tumors with a high proportion of cells in the over G2/M phase were significantly more likely to achieve radiological tumor shrinkage and disappearance of FDG uptake. This apparent discrepancy may be explained by the fact that aneuploid populations are not exclusively composed of cells in the Over G2/M phase but also contain cells in the G0/G1 phase. According to the Bergonie–Tribondeau law [30], cells with high proliferative activity and low differentiation are more radiosensitive. Aneuploid populations and cells in the Over G2/M phase may reflect such highly proliferative tumor subgroups, which could explain their increased sensitivity to radiation.

As for prognosis, although the difference did not reach statistical significance, patients with an Over G2/M ratio ≥ 2.16% tended to have better survival compared to those with an Over G2/M ratio < 2.16%. Prior studies have generally shown that aneuploid tumors are associated with poorer outcomes compared with diploid tumors [21]. Since Over G2/M cells are often included within aneuploid populations, one might expect poorer prognosis in patients with higher proportions of these cells. Interestingly, our findings suggest the opposite—that a higher proportion of Over G2/M phase cells may be associated with improved prognosis after radiation treatment. Whether this favorable prognosis is directly attributable to increased radiosensitivity warrants further investigation in a larger cohort.

The present findings suggest that flow cytometric analysis may contribute to treatment decision-making based on risk stratification in rectal cancer. In particular, patients with higher Over G2/M values may benefit from enhanced radiosensitivity, which could facilitate tumor shrinkage, secure surgical margins, and potentially improve outcomes following CRT. These observations highlight the potential role of cell-cycle profiling as a supplementary biomarker in guiding individualized therapeutic strategies.

This study has several limitations. First, this study was conducted at a single institution with a relatively small sample size. The lack of statistical significance for the prognostic impact of Over G2/M ≥ 2.16% may be attributable to the limited sample size and the relatively small number of events, which could have reduced the statistical power of the analysis. Second, there is a possibility of error in calculating the tumor shrinkage effect. 18F-FDG PET/CT was performed at a relatively early time point, 2–3 weeks after completion of CRT. At this stage, radiation-induced inflammation and edema may persist, leading to a favorable negative predictive value but a potentially diminished positive predictive value. Current guidelines recommend surgery 6–8 weeks after CRT to mitigate the impact of fibrosis [2]; thus, in view of planning subsequent surgical management, the timing of treatment evaluation was unavoidable. Third, intratumoral heterogeneity was not fully assessed, which may have influenced the interpretation of prognostic markers. In addition, partial shrinkage of the tumor after treatment and variability depending on the measurement site could have introduced errors in the assessment. These limitations should be considered when interpreting the present results, and further validation in a larger, prospective cohort is warranted.

## 5. Conclusions

The rapid flow cytometry analysis using Celltac PEAK may have potential utility in predicting the efficacy of CRT in rectal cancer. Cases with a high proportion of cells in the Over G2/M phase may have the potential for tumor shrinkage effects. Further validation is expected regarding the relationship between the cell cycle and the prediction of the effectiveness of CRT.

## Figures and Tables

**Table 1:** Patient demographics and comparison between the high Over G2/M group (≥2.16%) and the low Over G2/M group (<2.16%).

Patient Demographics	Over G2/M ≥ 2.16% (*n* = 15)	Over G2/M < 2.16% (*n* = 17)	*p* Value
Age, mean ± SD	62.9 ± 12.4	63.4 ± 9.95	0.910
Sex: male vs. female			1.00
male, *n* (%)	11 (73.3)	12 (70.6)	
Female, *n* (%)	4 (26.7)	5 (29.4)	
Location of the lesion			0.699
Rectosigmoid, Upper rectum, n (%)	5 (33.3)	4 (23.5)	
Lower rectum, n (%)	10 (66.7)	13 (76.5)	
Tumor size, cm, median (IQR)	4.3 (3.8–5.4)	4.7 (4.1–6.5)	0.249
Tumor invasion depth			1.000
cT2/T3, n (%)	10 (66.7)	11 (64.7)	
cT4, n (%)	5 (33.3)	6 (35.3)	
Lymph node metastasis			0.450
Negative, n (%)	6 (40.0)	4 (23.5)	
Positive, n (%)	9 (60.0)	13 (76.5)	
CEA, ng/mL, median (IQR)	4.9 (2.6–13)	5.2 (3.65–21.7)	0.265
CA19-9, mU/mL, median (IQR)	12.0 (0.9–18.9)	25.7 (11.4–66.5)	0.039 *
DNA Index, median (IQR)	1.68 (1.29–1.98)	1.36 (1.14–1.68)	0.220
Malignancy Index, median (IQR)	42.9 (28.0–64.0)	16.3 (9.49–38.2)	0.027 *
Aneuploidy			0.028 *
(+)	14	7	
(−)	1	10	
Treatment			0.486
CRT and inoperation for metastasis, n (%)	0 (0.00)	2 (11.8)	
CRT and operation, n (%)	15 (100)	15 (88.2)	
Follow-up period, months, median (IQR)	49.9 (17.8–60.0)	32.2 (6.65–58.0)	0.553
3 year recurrence			0.147
Recurrence-free, n (%)	12 (80)	9 (52.9)	
Recurrence, n (%)	3 (20)	8 (47.1)	

SD, standard deviation; CRT, chemoradiotherapy; IQR, interquartile range; cT2, clinical T2 stage; CEA, carcinoembryonic antigen; carbohydrate antigen 19-9; CRT, chemoradiotherapy; * Statistically significant difference.

**Table 2:** Tumor shrinkage in CT and cell cycle.

Patient Groups	CT Responder	CT Non-Responder	*p*
Cell cycle, %	Median (IQR)	Median (IQR)	
G0/G1	36.1 (20.4–47.8)	47.0 (37.2–60.0)	0.142
S	7.82 (3.26–26.5)	8.65 (5.90–20.0)	0.792
G2/M	4.95 (2.83–11.1)	3.02 (2.35–6.07)	0.152
Over G2/M	2.45 (1.14–4.41)	0.95 (0.27–2.08)	0.022 *
DNA Index	1.62 (1.37–2.08)	1.36 (1.19–1.70)	0.220
Malignancy Index	28.0 (10.4–62.0)	28.95 (13.4–39.4)	0.735
Aneuploidy, n			1.000
(+)	11	10	
(−)	5	6	

CT responder, the group with tumor shrinkage rate of 30% or more before and after CRT; CT non-responder, the group with less than 30% shrinkage; IQR, interquartile range; * Statistically significant difference.

**Table 3:** PET accumulation and cell cycle.

Patient Groups	Disappearance of PET Accumulation	Persistence of PET Accumulation	*p*
Cell cycle, %	Median (IQR)	Median (IQR)	
G0/G1	30.4 (17.1–44.9)	42.3 (20.9–60.7)	0.101
S	22.6 (6.64–34.5)	8.40 (4.89–20.1)	0.178
G2/M	9.57 (3.63–17.8)	3.35 (2.37–7.42)	0.111
Over G2/M	4.05 (2.29–4.36)	1.24 (0.45–2.34)	0.024 *
DNA Index	1.46 (1.20–1.75)	1.65 (1.22–1.96)	0.657
Malignancy Index	45.1 (29.9–64.1)	19.01 (9.82–42.9)	0.101
Aneuploidy, n			0.372
(+)	6	14	
(−)	1	9	

Disappearance of PET accumulation, the group in which the maximum standardized uptake value (SUV max) decreased to less than 5.0 after CRT; Persistence of PET accumulation, the group with SUV max equal to or above 5.0; IQR, interquartile range; * Statistically significant difference.

**Table 4:** Histological therapeutic grade and cell cycle.

Patient Groups	Therapeutic Grade ≥ 2	Therapeutic Grade ≤ 1	*p*
Cell cycle, %	Median (IQR)	Median (IQR)	
G0/G1	46.7 (27.3–61.4)	38.1 (24.8–47.7)	0.181
S	8.4 (4.94–24.5)	9.88 (6.46–21.0)	0.837
G2/M	3.26 (2.56–5.87)	5.75 (2.36–10.3)	0.537
Over G2/M	1.24 (0.38–2.62)	2.24 (0.98–4.32)	0.217
DNA Index	1.39 (1.22–1.51)	1.68 (1.19–1.82)	0.308
Malignancy Index	22.1 (14.2–60.0)	29.9 (11.7–44.1)	0.101
Aneuploidy, n			0.082
(+)	4	17	
(−)	5	4	

IQR, interquartile range.

**Table 5:** Hazard ratio for recurrence-free survival according to clinicopathologic variables. Cox proportional hazards regression analysis was performed to estimate hazard ratios and 95% confidence intervals (CIs) for each variable.

Variable	Recurrence-Free Survival	
	Hazard Ratio (95% CI)	*p* Value
Age	0.99 (0.94–1.05)	0.85
Sex: male vs. female	0.68 (0.20–2.35)	0.55
Location of the lesion: Rb vs. RS, Ra	1.82 (0.39–8.45)	0.41
Tumor size, cm	1.01 (0.97–1.04)	0.45
Tumor invasion depth: cT4 vs. cT2/T3	0.61 (0.16–2.30)	0.45
Lymph node metastasis: (+) vs. (−)	5.23 (0.66–40.9)	0.04 *
CEA, ng/mL	1.02 (1.00–1.04)	0.03 *
CA19-9, mU/mL	1.00 (1.00–1.00)	0.01 *
Cell cycle, %		
G0/G1	1.00 (0.97–1.04)	0.59
S	1.00 (0.96–1.04)	0.70
G2/M	1.00 (0.89–1.07)	0.97
Over G2/M	0.76 (0.49–1.06)	0.11
DNA Index	0.18 (0.001–5.08)	0.34
Malignancy Index	1.00 (0.97–1.03)	0.61
Aneuploidy: (+) vs. (−)	0.35 (0.10–1.16)	0.08

CI, Confidence interval; cT2, clinical T2 stage; CEA, carcinoembryonic antigen; carbohydrate antigen 19-9; * Statistically significant difference.

## Data Availability

The raw data supporting the conclusions of this article will be made available by the authors on request.
